# The potential role of *N*^7^-methylguanosine (m7G) in cancer

**DOI:** 10.1186/s13045-022-01285-5

**Published:** 2022-05-19

**Authors:** Yuejun Luo, Yuxin Yao, Peng Wu, Xiaohui Zi, Nan Sun, Jie He

**Affiliations:** 1 Department of Thoracic Surgery, National Cancer Center/National Clinical Research Center for Cancer/Cancer Hospital, Chinese Academy of Medical Sciences and Peking Union Medical College, Beijing, 100021 China; 2 State Key Laboratory of Molecular Oncology, National Cancer Center/National Clinical Research Center for Cancer/Cancer Hospital, Chinese Academy of Medical Sciences and Peking Union Medical College, Beijing, China

**Keywords:** *N*^7^-methylguanosine (m7G), RNA modification, RNA metabolism, Cancer

## Abstract

*N*^7^-methylguanosine (m7G), one of the most prevalent RNA modifications, has recently attracted significant attention. The m7G modification actively participates in biological and pathological functions by affecting the metabolism of various RNA molecules, including messenger RNA, ribosomal RNA, microRNA, and transfer RNA. Increasing evidence indicates a critical role for m7G in human disease development, especially cancer, and aberrant m7G levels are closely associated with tumorigenesis and progression via regulation of the expression of multiple oncogenes and tumor suppressor genes. Currently, the underlying molecular mechanisms of m7G modification in cancer are not comprehensively understood. Here, we review the current knowledge regarding the potential function of m7G modifications in cancer and discuss future m7G-related diagnostic and therapeutic strategies.

## Introduction

Owing to the continuous development of high-throughput sequencing technology, posttranscriptional modifications have been identified to play a pivotal role in a variety of physiological and pathological processes [1–3]. To date, more than 170 types of RNA modifications have been discovered in different molecules, including messenger RNA (mRNA), ribosomal RNA (rRNA), transfer RNA (tRNA), and long noncoding RNA (lncRNA) [1, 4, 5]. *N*^7^-methylguanosine (m7G) modification, one of the most prevalent RNA modifications, is often located at the 5’ caps and internal positions of eukaryotic mRNA or internally within rRNA and tRNA of all species [6–12]. Furthermore, recent studies have uncovered that m7G methylation also occurs in microRNA (miRNA) [13, 14]. Meanwhile, methods for the detection of m7G modifications are being continuously updated and include m7G-MeRIP-Seq, m7G-Seq, and m7G-miCLIP-Seq techniques [8, 15]. The conventional m7G-MeRIP-Seq approach relies on antibody immunoprecipitation and has limited resolution (~ 80 bp) [15]. Based on the termination of reverse transcription, m7G-Seq achieves base resolution in the detection of m7G modification in human mRNA and tRNA [15]. The novel m7G-miCLIP-Seq has higher sensitivity and specificity in the mapping of m7G modifications through a modified miCLIP protocol [8].

In mammals, the most well-studied regulator of m7G is methyltransferase-like 1 (METTL1), which binds to its corresponding cofactor WD repeat domain 4 (WDR4) to install m7G modifications in tRNA, miRNA, and mRNA [7]. RNA guanine-7 methyltransferase (RNMT) and its cofactor RNMT-activating miniprotein (RAM) actively participate in m7G modification installment at the 5’ caps of mRNA [16]. Williams–Beuren syndrome chromosome region 22 (WBSCR22) and tRNA methyltransferase activator subunit 11–2 (TRMT112) are responsible for mediating m7G methylation in rRNA [17]. Since m7G methylation can mediate RNA metabolism and function, it is thought to act as a molecule handler for the alteration of target gene expression [6, 7, 18, 19]. It has been reported that m7G modification participates in various disorders, including aberrant stem cell growth and differentiation, Galloway Mowat syndrome, microcephalic primordial dwarfism, and teratoma formation [20–23].

Some evidence demonstrates that m7G methylation is closely associated with tumor development and is involved in multiple tumor-related biological activities [24–27]. In the present review, we concentrate on the cutting-edge progress made in the study of m7G modification with a particular focus on the functions of m7G in cancer development and progression, in addition to exploring the future directions of m7G research.

### Regulators of m7G

**Fig. 1:**
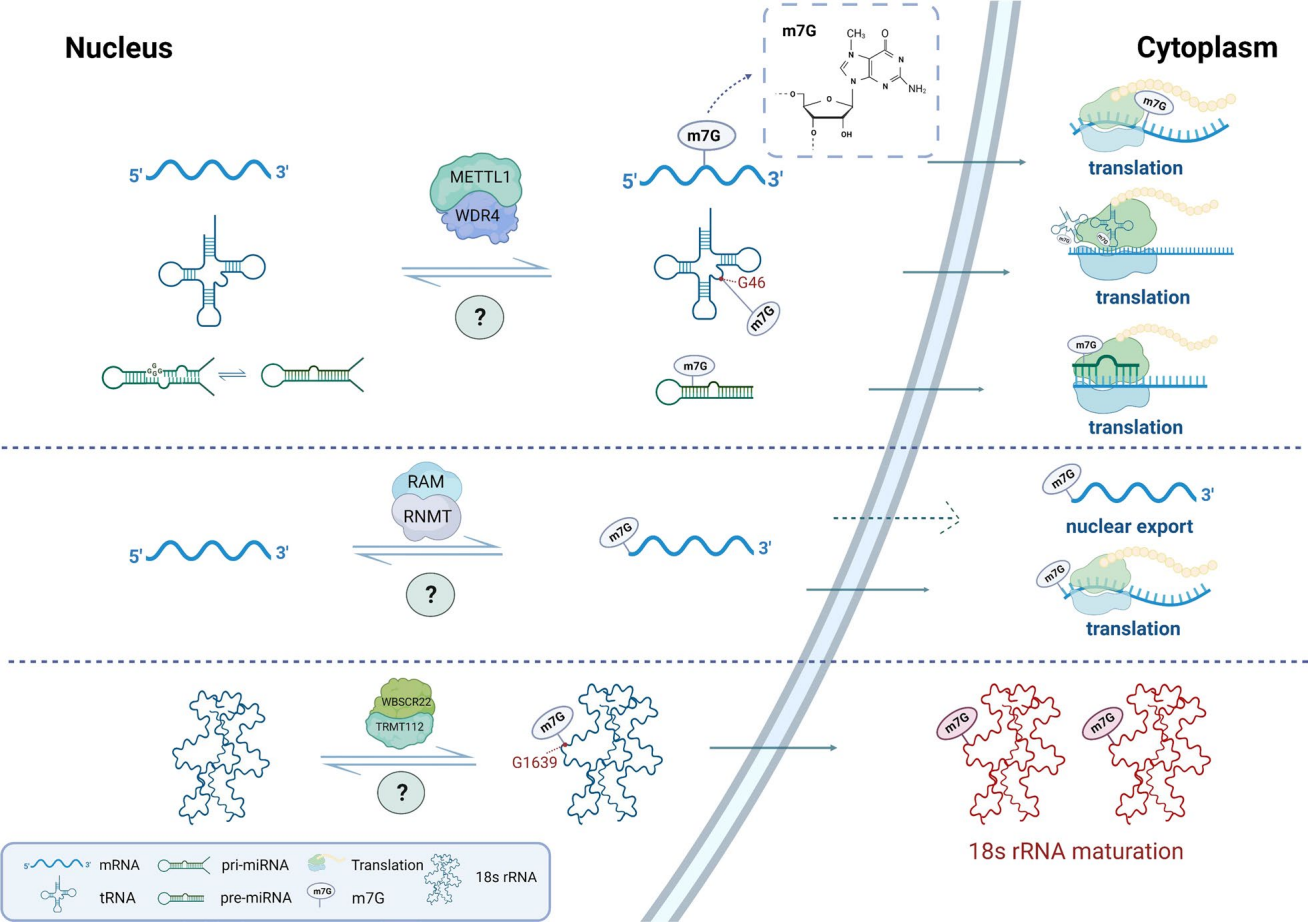
The cellular m7G modification machinery. The m7G modification is a multilayered process. The m7G methyltransferases have been identified as METTL1, WDR4, RNMT, RAM, WBSCR22, and TMRT112, which install the m7G modification on target RNA molecules, including mRNA, tRNA, rRNA, and miRNA. The METTL1/WDR4 complex installs m7G modification on mRNA (internal site), tRNA (G46 site), and miRNA (G-quadruplex structure), ultimately regulating global translation. The RNMT/RAM complex is responsible for installation of the m7G modification on the 5’ cap of mRNA, mediating its nuclear export and translation processes. The WBSCR22/TMRT112 complex adds m7G modification at the G1638 site of 18 s rRNA, which facilitates 18 s rRNA maturation

Currently, the understanding of the regulators of m7G modification is still at a preliminary stage. Identified m7G regulators include the Trm8p/Trm82p heterodimeric complex and Bud23/Trm112 in yeast, and the corresponding orthologs in mammals, METTL1/WDR4 and WBSCR22/TRMT112 [7]. In addition, RNMT and RAM also participate in m7G modification in mammals [16]. Here, we focus on m7G modification in humans, the regulators of which are all m7G methyltransferases whose main function is to add m7G modification to target RNAs, thereby affecting the production, structure, and maturation of RNA, and ultimately mediating a variety of critical biological processes (Fig. 1).

### METTL1/WDR4

The best-characterized m7G regulators are METTL1 and WDR4, which form a complex to catalyze the m7G modification of various types of RNAs. The METTL1/WDR4 complex enables the installation of m7G modifications at the G46 site of various tRNA variable loops, stabilizing the tRNA tertiary structure [7, 22, 28]. Meanwhile, this METTL1/WDR4 complex is indispensable for normal mRNA translation and neural self-renewal and lineage differentiation [20]. METTL1/WDR4 complex deficiency in mouse embryonic stem cells significantly dysregulates the cell cycle and proliferation by impacting tRNA function and the translation of various mRNAs [20]. Furthermore, METTL1 is also involved in the pluripotency of human stem cells by regulating the translation of various stem cell markers, including octamer-binding transcription factor 4 (*OCT4*), Nanog homeobox (*NANOG*), and SRY-Box transcription factor 2 (*SOX2*) [23]. Silencing METTL1 not only impairs the pluripotency and cell cycling of human stem cells but also facilitates teratoma development [23].

Additionally, METTL1/WDR4 complex-dependent m7G modification also occurs in miRNA. METTL1/WDR4 promotes miRNA biogenesis by adding the m7G modification to the G-quadruplex structure of the primary miRNA transcript (pri-miRNA) [13]. In A549 cells, depletion of METTL1 leads to a decrease in let-7 miRNA processing and promotion of cell migration [13].

Moreover, METTL1/WDR4 has been identified as the m7G writer of mRNA. METTL1 functions as an m7G methyltransferase to install the m7G modification in target mRNA, while WDR4 facilitates the binding of the heterodimer complex to the target mRNA [15]. This METTL1/WDR4-dependent m7G modification of mRNA is related to translation efficacy. In HeLa cells, both transient and stable knockdown of METTL1 significantly decreases the translation efficacy of mRNAs that bear METTL1-dependent m7G modifications [15]. METTL1 has been shown to increase the translation of vascular endothelial growth factor A (*VEGFA*) mRNA in an m7G-dependent manner, thereby stimulating post-ischemic angiogenesis [29].

### WBSCR22/TRMT112

The m7G modification in 18S rRNA is installed by the WBSCR22/TRMT112 complex in humans and the Bud23/Trm112 complex in yeast [30–32]. The WBSCR22/TRMT112 methyltransferase complex installs the m7G modification at the specific G1639 location in 18S rRNA, which actively participates in 18S rRNA precursor biogenesis and is also needed for nuclear export of the 40S rRNA subunit regardless of its catalytic activity [17, 30]. Inhibition of WBSCR22 has been shown to intensify the accumulation of 18S rRNA precursors in the nucleus, which eventually attenuates 18S rRNA maturation [17, 33].

### RNMT/RAM

RNMT and RAM form an enzyme complex that installs m7G modifications. RAM is responsible for stabilizing the RNMT structure and recruiting target RNA [34]. They cooperate to produce an m7G-modified 5’ cap in mRNA, 5’m7GpppX, which not only protects RNA from exonuclease cleavage, but also influences RNA processing, export, and translation [35]. The eukaryotic translation initiation factor 4E (*EIF4E*) specifically and directly binds to this m7G cap, ultimately influencing target RNA export and translation efficiency [36]. A previous study also observed that enhanced expression of RNMT/RAM promotes Cyclin D1 (*CCND1*) translation by increasing m7G capping of its mRNA, which ultimately facilitates mammary epithelial and fibroblast cell transformation [37].

### Cross talk between m7G modification and other posttranscriptional modifications

**Fig. 2:**
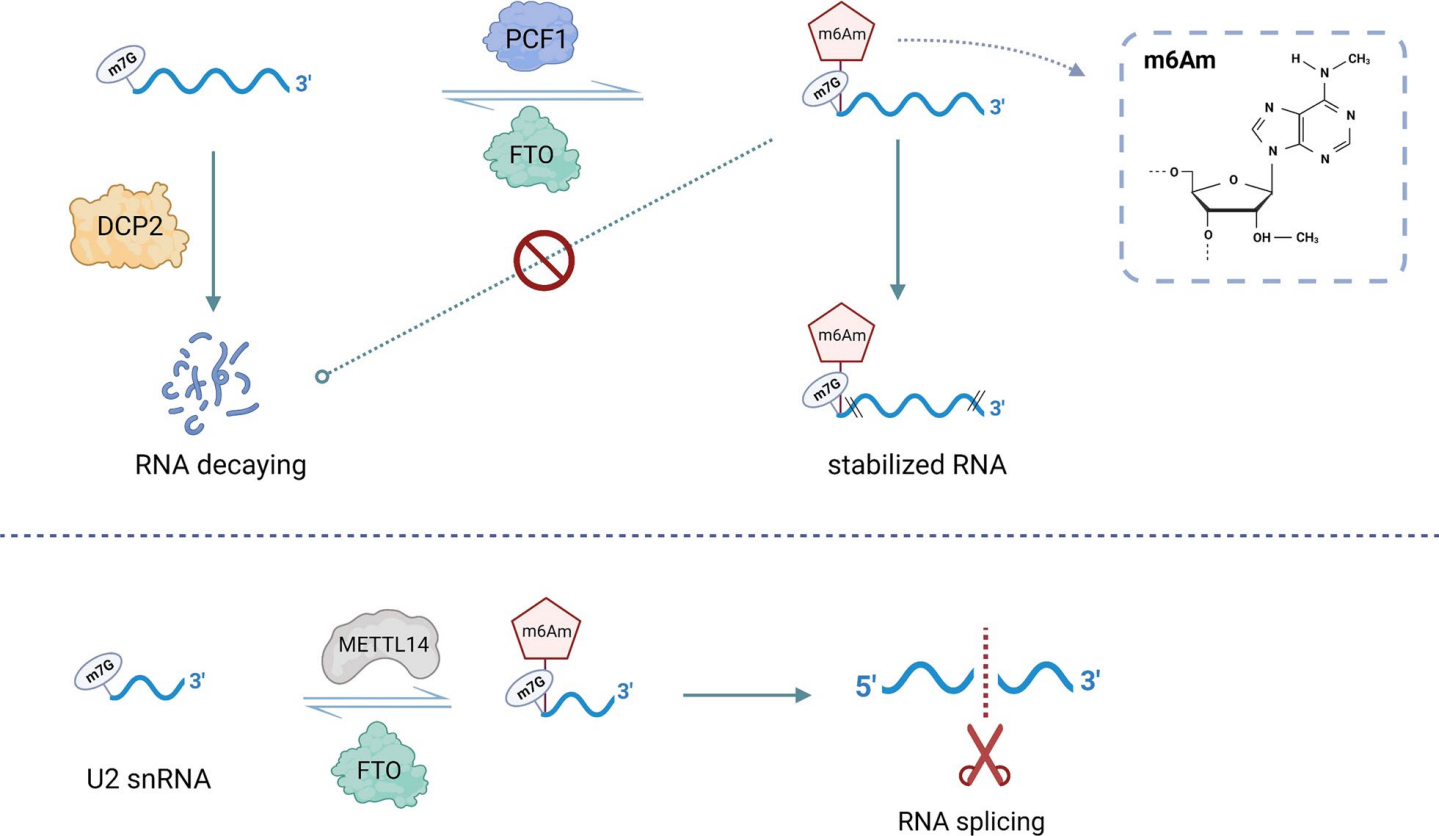
The cross talk between m7G modification and other posttranscriptional modifications. The m6Am methyltransferase PCIF1 catalyzes m6Am modification adjacent to the m7G cap, which enhances resistance to DCP2-dominated decapping. The m6Am methyltransferase METTL14 installs m6Am modification at an internal site in U2 snRNA, ultimately influencing global alternative RNA splicing

It is well known that posttranscriptional regulation is intricate, with multiple interconnected posttranscriptional regulators that function together. m7G modification, a novel research topic, is still at a preliminary stage; however, existing studies have demonstrated that m7G cooperates with other RNA modifications in different biological processes (Fig. 2). The decay of mRNA containing m7G-modified 5’ caps is regulated by mRNA-decapping enzyme 2 (*DCP2*) [38]. The *N6*, *2′-O*-dimethyladenosine (m6A_m_) modification is also involved in the regulation of mRNA decay [39]. If the first nucleotide after the m7G modification is *2′-O*-dimethyladenosine (A_m_), the A_m_ site is often catalyzed by m6A_m_ methyltransferase phosphorylated CTD interacting factor 1 (*PCIF1*) to form an m6A_m_ modification. Meanwhile, FTO also enables removal of such m6A_m_ modifications, maintaining a dynamic and reversible m6A_m_ modification process Fig. 2. The m7G cap adjacent to the m6A_m_ modification protects mRNA from decapping by DCP2 [39]. Moreover, m6A_m_ modifications have also been found at internal sites of U2 small nuclear RNA (snRNA) containing an m7G-modified cap, which is added by another m6A_m_ methyltransferase, methyltransferase-like 14 (*METTL14*) [40]. The internal m6A_m_ modification and m7G cap cooperate to regulate global RNA alternative splicing, which may further influence more biological functions in human diseases [40].

### Role of m7G in cancer

**Fig. 3:**
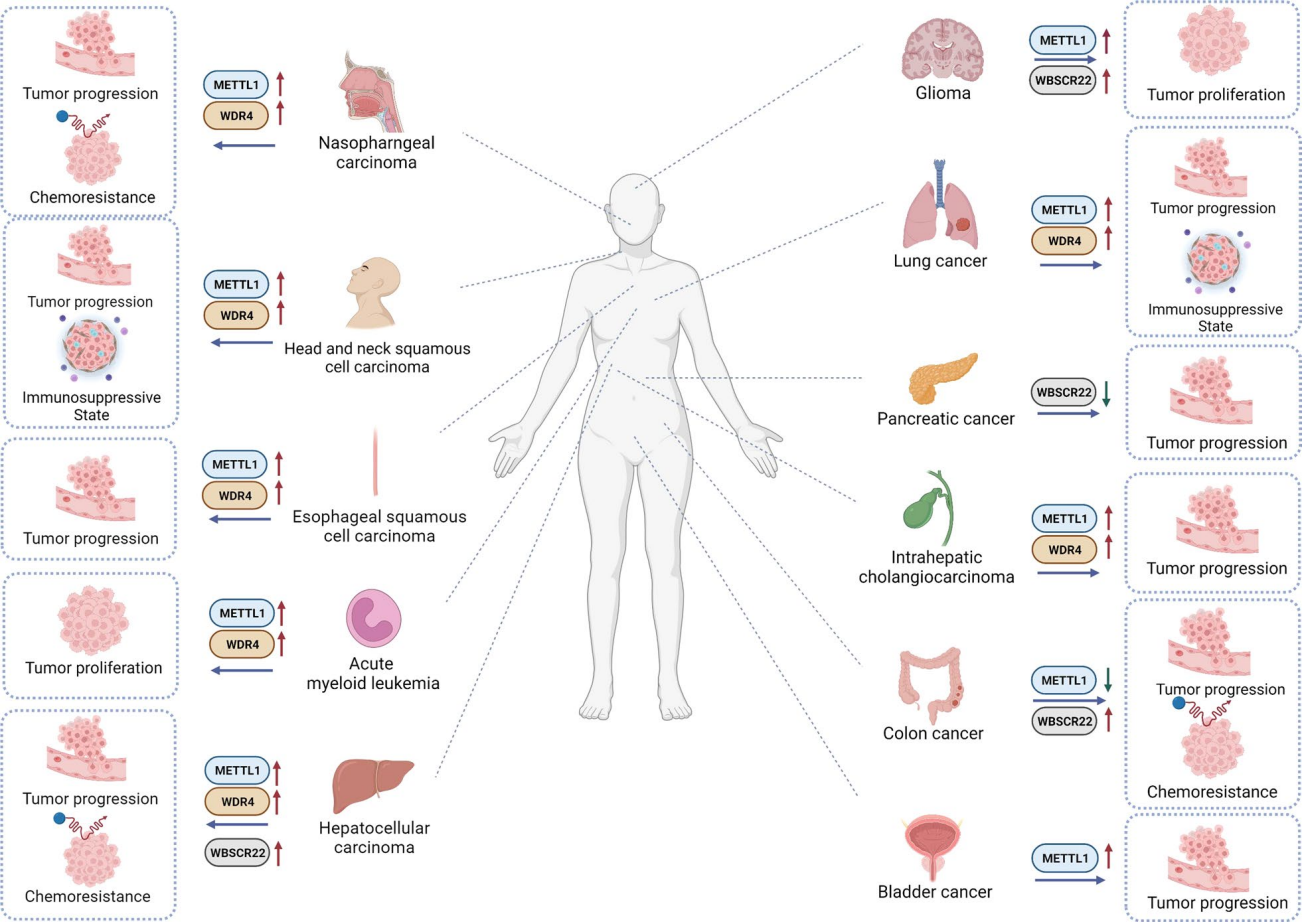
The role of m7G regulators in various tumors. The m7G regulators are involved in tumor development and progression in different cancers, including AML, BC, CC, ESCC, glioma, HCC, HNSCC, ICC, LC, NPC, and PC. These m7G regulators play a dual role in various cancers, promoting or inhibiting tumor progression by regulating the expression of tumor-related genes

To date, a variety of studies have demonstrated that m7G modification is significantly involved in tumorigenesis and the progression of various cancers. The m7G methyltransferases are often aberrantly expressed in cancers and catalyze m7G modification in tRNA or miRNA, ultimately influencing target gene expression and regulating tumor-related biological functions. Recent studies have revealed the expression levels of m7G regulators and their underlying molecular mechanisms in tumors (Table 1). Here, we summarize the potential roles of the m7G modification in various cancers (Fig. 3).

**Table 1:** The roles of m7G regulators and underlying molecular mechanisms in cancer

Cancer types	m7G regulators	Expression	Molecular axis	Function	Role in cancer	References
AML	METTL1 WDR4	Upregulation Upregulation	METTL1/WDR4/Arg-TCT-4-1/cell cycle genes	Promoting cell growth, proliferation, and tumor formation	Oncogene Oncogene	[43]
BC	METTL1	Upregulation	METTL1/EGFR/EFEMP1	Promoting cell proliferation, migration, and invasion	Oncogene	[26]
CC	METTL1	Downregulation	METTL1/let-7e miRNA/HMGA2	Inhibiting cell proliferation, migration, and invasion.	Anti-oncogene	[48]
	WBSCR22	Upregulation	METTL1/miR-149-3P/P53/S100A4	Promoting cell sensitivity to cisplatin	Oncogene	[49]
				Promoting cell apoptosis and resistance to oxaliplatin		[50]
ESCC	METTL1 WDR4	Upregulation Upregulation	METTL1/WDR4/RPTOR/ULK1/autophagy pathway	Promoting cell proliferation and tumor formation	Oncogene Oncogene	[52]
Glioma	METTL1	Upregulation	METTL1/MAPK pathway	Promoting cell growth and proliferation	Oncogene	[56]
	WBSCR22	Upregulation	PI3K/AKT/GSK3β pathway	Promoting cell growth and metastasis	Oncogene	[57]
HCC	METTL1	Upregulation	METTL1/WDR4/PTEN pathway	Promoting cell proliferation and migration	Oncogene	[60]
			METTL1/WDR4/Cyclin A2/EGFR/VEGFA	Promoting cell growth, invasion, and migration	Oncogene	[24]
	WDR4	Upregulation	WDR4/EIF2A/CCNB1	Promoting cell growth, migration, and resistance to sorafenib	Oncogene	[61]
HNSCC	METTL1	Upregulation	METLL1/WDR4/ PI3K/AKT/mTOR pathway	Promoting cell growth, migration, and invasion	Oncogene	[59]
	WDR4	Upregulation			Oncogene	
ICC	METTL1	Upregulation	METTL1/WDR4/cell cycle and EGFR-related genes	Promoting cell growth, proliferation, migration, and invasion	Oncogene	[66]
	WDR4	Upregulation			Oncogene	
LC	METTL1	Upregulation	METTL1/WDR4/CCND3/CCNE1	Promoting cell proliferation, migration, and invasion	Anti-oncogene/oncogene	[68]
			METTL1/AKT/mTORC1 pathway	Promoting cell proliferation and autophagy		[69]
			METTL1/let-7 miRNA	Inhibiting cell migration		[13]
	WDR4	Upregulation	WDR4/PML/uPAR/SAA2/MMP2/MMP9	Promoting cell metastasis	Oncogene	[70]
NPC	METTL1	Upregulation	METTL1/WDR4/WNT/EMT pathway	Promoting cell proliferation, migration, and invasion	Oncogene	[78]
	WDR4	Upregulation			Oncogene	
PC	WBSCR22	Downregulation	WBSCR22/TRMT112/ISG15	Inhibiting cell proliferation, invasion, and tumor formation	Anti-oncogene	[80]

*AML* acute myeloid leukemia, *BC* bladder cancer, *CC* colon cancer, *ESCC*, esophageal squamous cell carcinoma*, HCC* hepatocellular carcinoma, *HNSCC,* head and neck squamous cell carcinoma*, ICC* intrahepatic cholangiocarcinoma, *LC* lung cancer, *NPC* nasopharyngeal carcinoma, *PC* pancreatic cancer

### Acute myeloid leukemia

Acute myeloid leukemia (AML) is the leading type of acute leukemia in adults, with an incidence greater than 80% in this population [41]. AML features a high level of genomic aberrations and molecular heterogeneity [42]. The expression of METTL1 and WDR4 is highly increased in AML patient samples at both the mRNA and protein levels [43]. Moreover, stable knockdown of METTL1 effectively suppresses leukemic stem cell growth but has a negligible influence on normal isogenic hematopoietic stem and progenitor cells [43]. METTL1-knockout mice exhibit lower tumor burden and longer survival than wild-type controls. Mechanistically, the METTL1/WDR4 complex increases the abundance of tRNA with m7G modifications, especially tRNA-Arg (anticodon TCT) 4–1 (Arg-TCT-4–1), which affects the translation of mRNA enriched with AGA codons by decreasing ribosome pausing efficacy, in particular cell cycle progression genes. Therefore, METTL1/WDR4-dominated m7G modification of tRNA drives the pathogenesis process through remodeling of mRNA translation activities to enhance the expression of cell cycle progression genes, which may represent a novel target for AML treatment.

### Bladder cancer

Bladder cancer (BC), the ninth most widespread cancer globally, is a severe health issue with increasing mortality every year [44]. Despite advances in surgery and adjuvant treatment, approximately 50% of patients with BC die from tumor metastasis [45]. There has been little advancement in therapeutic strategies for BC over recent decades [46]. METTL1 is highly upregulated in BC tissues, and its expression is positively correlated with advanced clinical stage and high tumor grade [26]. METTL1 plays an oncogenic role in tumorigenesis and progression in BC. Functional experiments have demonstrated that depletion of METTL1 effectively limits BC proliferation, migration, and invasion both in vitro and in vivo. In BC, METTL1 mediates specific mRNA translation (*EGFR* and EGF-containing fibulin extracellular matrix protein 1 (*EFEMP1*)) by altering the m7G modification of tRNA and restraining ribosome pausing during tRNA–mRNA codon identification. The important role of METTL1 in BC tumor progression and its value in clinicopathology provides potential ideas for the clinical management and treatment of BC.

### Colon cancer

Colon cancer (CC) is the most widespread digestive malignancy worldwide [47]. Evaluation of METTL1 expression levels by real-time quantitative polymerase chain reaction (qPCR) and western blotting demonstrated significant downregulation in CC tissues as compared with normal tissues [48]. It was discovered that METTL1 acts as a tumor suppressor in CC, overexpression of which can effectively attenuate the proliferation, migration, and invasion of CC cells, in addition to promoting apoptosis [48]. Further findings suggest that METTL1 positively regulates let-7e miRNA expression to mediate the downstream target gene high mobility group AT-hook 2 (*HMGA2*). It is clear that the METTL1/let-7e miRNA/HMGA2 axis is closely related to the development of CC; nevertheless, METTL1 is also involved in the chemosensitivity of cisplatin [49]. The sensitivity of CC cells to cisplatin is remarkably enhanced following overexpression of METTL1. Underlying this process is the promotion of miR-149-3p expression by METTL1, which cooperates to increase *P53* expression and decrease S100 calcium-binding protein A4 (*S100A4*) expression at both the mRNA and protein levels [49]. METTL1 actively participates in multiple tumor progression-related processes in CC by regulating the expression levels of various miRNAs, which may provide insight into novel therapeutic targets for CC [48, 49].

WBSCR22 is also significantly overexpressed in CC and acts as a predictive factor for poor patient survival. Depletion of WBSCR22 can activate oxaliplatin-induced cellular apoptosis, significantly decreasing resistance to oxaliplatin in CC [50].

### Esophageal squamous cell carcinoma

Esophageal squamous cell carcinoma (ESCC) is highly susceptible to lymph node metastasis and vascular invasion, and patients with ESCC are often diagnosed at an advanced stage, creating the need for the development of additional treatment strategies [51]. A recent study revealed that the expression of METTL1 and WDR4 is aberrantly upregulated in ESCC, which is also associated with inferior clinical outcomes in patients with ESCC [52]. Inhibition of METTL1 or WDR4 is an effective method to decrease ESCC progression both in vitro and in vivo, including impairing tumor proliferative capacity and tumor formation. METTL1 and WDR4 negatively regulate ribosome pausing at m7G-related codons, ultimately leading to a significant decrease in the level of transcription of negative regulators of autophagy-related pathways (regulatory-associated protein of mTOR (*RPTOR*) and Unc-51-like autophagy-activating kinase 1 (*ULK1*)). The *METTL1* knockout and knockin mice further unveiled the oncogenic function of METTL1 in ESCC tumorigenesis and progression. The METTL1/WDR4/RPTOR/ULK1/autophagy axis may provide potential therapeutic strategies for ESCC treatment.

### Glioma

Glioma is the most frequent central nervous system tumor and is characterized by high recurrence and mortality rates [53, 54]. METTL1 is overexpressed in glioma as compared with adjacent normal tissues and increases with increasing tumor grades. METTL1 also demonstrates a high level of genomic amplification in glioma [55]. METTL1 enhances the proliferation and growth of glioma, which may involve the tumor-related MAPK signaling pathway [56]. WBSCR22 is also upregulated in glioma and acts as an unfavorable survival predictor [57]. WBSCR22 enhances glioma cell growth and metastasis by regulating the PI3K/AKT/GSK3β signaling pathway. Loss of WBSCR22 can decrease the phosphorylation of AKT and GSK3β, which also destabilize the intracellular levels of *CCND1* and β-catenin, thus inhibiting glioma progression. These findings may shed some light on glioma treatment.

### Head and neck squamous cell carcinoma

Head and neck squamous cell carcinoma (HNSCC) is featured by rapid growth, regional lymphatic spread, and a bleak prognosis [58]. The upregulated expression of METTL1 and WDR4 actively drives HNSCC development and progression [59]. Patients with higher expression levels of METTL1 and WDR4 tend to suffer worse prognoses than their lower expression counterparts. These two m7G regulators facilitate HNSCC cell growth, migration, and invasion, while inhibiting HNSCC cell apoptosis. Ablation of METTL1 reduces the ribosomal transition efficiency of m7G-modified tRNA-decoding codons by increasing ribosome pausing. The expression levels of genes enriched in the PI3K/AKT/mTOR pathway are the most influenced in a METTL1-related m7G-tRNA-dependent manner, including *PI3K*, *CCND1*, vimentin, matrix metallopeptidase 9 (*MMP9*), B cell lymphoma-2 (*Bcl-2*), and phosphorylation of S6 kinase (*P-S6K*). Notably, METTL1 is involved in reshaping the TIME. *METTL1* knockout mice have an anti-tumor microenvironment, with higher infiltration of CD4 + memory T cells, CD4 + naïve T cells, and CD8 + naïve T cells, and lower infiltration of Tregs and Th17 cells. Meanwhile, a tumor-promoting interaction (interleukin 1 beta (*IL1β*)–interleukin A receptor type 2 (*IL1R2*)) between cancer cells and macrophages is also suppressed in METTL1-knockout mice. Taken together, these findings provide fundamental evidence for METTL1/WDR4-related therapeutic strategies for HNSCC.

### Hepatocellular carcinoma

Hepatocellular carcinoma (HCC) is regarded as the sixth most prevalent tumor worldwide, with approximately 841,000 new cases and 782,000 deaths annually [47]. m7G has shed new light on the inexplicit molecular mechanism of HCC and could possibly direct new therapeutic strategies. Several studies have analyzed the expression levels of METTL1 and WDR4 based on data available in public databases and reported upregulation in HCC samples as compared with normal liver tissue [60, 61]. The high expression of METTL1 and WDR4 is not only related to advanced tumor stage and grade but also to poor clinical outcome in patients with HCC. The oncogenic function of METTL1 in HCC has been reported to boost proliferation and migration by suppressing PTEN-related signaling pathways [60]. Another study validated the high protein expression levels of METTL1 and WDR4 in HCC tissues versus corresponding normal tissues and demonstrated that the inhibition of METTL1 or WDR4 globally decreases m7G tRNA modification and reduces HCC progression [24]. With respect to the mechanism, METTL1/WDR4 regulates the translation of cyclin A2 (*CCNA2*), epidermal growth factor receptor (*EGFR*), and vascular endothelial growth factor A (*VEGFA*) mRNA in an m7G-modified tRNA-dependent manner. Notably, METTL1-knockout mice exhibit reduced hepatocarcinogenesis as compared with control mice, showing slower tumor formation and reduced tumor burden.

It has been revealed that WDR4 enhances various malignant phenotypes of HCC [61]. WDR4 reduces apoptosis of HCC cells by increasing G2/M cell cycle transition, while intensifying metastasis and sorafenib resistance by impacting the epithelial–mesenchymal transition (EMT) process. Mechanistically, WDR4 promotes the transcription of Cyclin B1 (*CCNB1*) by facilitating the binding of eukaryotic translation initiation factor 2A (*EIF2A*) to CCNB1 mRNA transcripts, while WDR4 itself is also regulated by *MYC*. Taken together, m7G regulators serve an indispensable function in the development of HCC, which may provide potential targets for future treatment.

The promoters of WBSCR22 and RNMT are hypomethylated in HCC as compared with adjacent normal tissue [62]. Inhibition of WBSCR22 or RNMT has an anticancer effect on HCC, including the cessation of proliferation and invasion [62].

### Intrahepatic cholangiocarcinoma

Intrahepatic cholangiocarcinoma (ICC) is one of the most fatal digestive tumors, with a five-year survival rate of only 5–40% [63, 64]. Novel therapeutic strategies are awaited due to the lack of thorough comprehension of tumorigenesis and effective approaches to limit the outgrowth of ICC [65]. A recent study uncovered the function of m7G modification and the corresponding regulators in ICC [66]. Firstly, the expression of METTL1 and WDR4 is significantly increased at both the mRNA and protein levels in ICC as compared with that in peri-tumor tissues, and these also act as poor survival predictors in ICC patients. METTL1 and WDR4 carry out tumor-promoting functions, enhancing the growth, migration, and invasion of ICC cells. The underlying molecular mechanism is METTL1/WDR4-regulated specific oncogenic mRNA translation performed in an m7G-modified tRNA-dependent manner, which reduces ribosome pausing at m7G-modified tRNA-decoded codons. METTL1/WDR4 influences the m7G modification and expression of specific tRNAs in a codon frequency-dependent manner, thus modulating the translation of oncogenic mRNA including cell cycle (CCNA2, CCND2, CDK6, and CDK8) and EGFR pathways (EGFR, AKT, and mTOR) genes. This sheds some light on future novel targets for ICC treatment.

### Lung cancer

Lung cancer (LC) displays the highest incidence and mortality among all malignancies worldwide [67]. In both lung adenocarcinoma and squamous carcinoma, the expression levels of METTL1 and WDR4 are significantly elevated as compared with those in normal lung tissues and are closely associated with an unfavorable prognosis in patients with LC [68]. Recent studies have revealed that METTL1 and WDR4 play oncogenic roles in the development of LC and are essential for the growth, migration, and invasion of LC cells both in vitro and in vivo [68]. Moreover, METTL1 depletion not only reduces the expression of tRNA with m7G modifications but also inhibits the translation efficiency of mRNA with a high m7G composition in LC cells. Furthermore, two cell cycle regulators (*CCND3* and *CCNE1*) have been shown to be downstream targets of METTL1/WDR4 and facilitate LC progression [68]. Another study also suggested a tumor promotor role of METTL1 in LC by demonstrating its ability to accelerate proliferation and autophagy through the AKT/mTORC1 signaling cascade [69]. Nevertheless, contradictory results have shown that overexpression of METTL1 can effectively impair the migratory ability of LC A549 cells by mediating let-7 miRNA biogenesis and expression, and inhibition of METTL1 expression can significantly enhance migratory ability [13]. Further in-depth research is required to reveal the complex function of METTL1 in LC.

A previous study reported that WDR4 promotes the proteasomal degradation of promyelocytic leukemia (*PML*) tumor suppressor in LC [70]. The WDR4/PML axis increases the expression of various oncogenic factors including CD73, urokinase plasminogen activator surface receptor (*uPAR*), serum amyloid A2 (*SAA2*), *MMP2*, and matrix metallopeptidase 9 (*MMP9*), which are known to promote metastatic phenotypes [71–74]. Furthermore, the WDR4/PML cascade enhances immunosuppressive cell infiltration (Tregs and M2 macrophages) while impairing CD8 + T cell infiltration. WDR4 negatively regulates *PML* expression to enhance LC development by creating a pro-metastatic and immunosuppressive status, which may be helpful for potential future treatments in LC patients.

### Nasopharyngeal carcinoma

Nasopharyngeal carcinoma (NPC) is an urgent public health burden in east and southeast Asia [75]. Unfortunately, most cases are already at an advanced stage at diagnosis, and there are limited treatment methods for advanced cases after disease relapse and progression [76, 77]. The latest research has identified the oncogenic role of METTL1/WDR4 in NPC [78]. Both METTL1 and WDR4 are elevated in NPC, which is also positively correlated with a worse outcome for patients with NPC. The inhibition of METTL1 and WDR4 weakens tumorigenesis of NPC, which significantly restrains the tumor growth, migratory, and invasion features, and increases cell apoptosis both in vivo and in vitro. Functional exploration has revealed that METTL1/WDR4 actively modulates tRNA m7G modification, which influences global mRNA translation by decreasing ribosome pausing at m7G-tRNA-dependent codons recognition process. Notably, the classical oncogenic pathways (WNT/β-catenin and EMT) are mediated by the METTL1/WDR4 complex, and critical pathway-related mRNA expression is determined by METTL1/WDR4-dominated m7G modification of tRNA machinery. Moreover, METTL1 aggravates resistance to cisplatin and docetaxel in NPC cells via the WNT signaling pathway, and depletion of METTL1 can effectively revive the chemosensitivity of NPC cells. Collectively, these findings may provide new insight into molecular-targeted therapies for patients with NPC.

### Pancreatic cancer

Pancreatic cancer (PC) is notorious for its aggressive and fatal nature, with a 5-year survival rate of only 8% [79]. A recent study explored the function of WBSCR22 and TRMT112 in PC, and it was found that WBSCR22 is downregulated in PC samples as compared with surrounding normal pancreatic tissue and is related to longer survival times in PC patients [80]. WBSCR22 works in concert with TRMT112 to exert a tumor suppressor effect in PC, which negatively mediates translation of the oncogenic factor interferon-stimulated gene 15 (*ISG15*). ISG15 is significantly upregulated in PC specimens and favors various malignant phenotypes (rapid cell proliferation, invasion, and tumor formation) of PC. Meanwhile, preclinical experiments have shown that overexpression of WBSCR22 and TRMT112 significantly decreases malignant phenotypes by downregulating ISG15 expression in PC. The WBSCR22/TRMT112/ISG15 axis may be an innovative strategy for PC therapy in the future.

### Other cancers

Since m7G-related tumor studies are still in the preliminary stage, the remaining tumor types are grouped together in this section. Several studies have revealed upregulated expression of METTL1 in other tumors, including invasive breast carcinoma (BRCA), kidney renal clear cell carcinoma (KIRC), prostate adenocarcinoma, rectal carcinoma, and uterine corpus endometrial carcinoma [55, 56, 81, 82]. Additionally, METTL1 exhibits a high level of genomic amplification in various tumors including sarcoma and adrenocortical carcinoma [55]. METTL1 is associated with a poor prognosis in KIRC and mesothelioma patients, while it is related to favorable survival in patients with ovarian serous cystadenocarcinoma [56]. Based on data from functional analysis, METTL1 is likely to interrelate and function with various RNA regulators and DNA packaging complexes in human tumors [55]. Additionally, METTL1 is associated with sensitivity to 5-fluorouracil, and stable knockdown of METTL1 in HeLa cells effectively increases its cytotoxic effect [83]. Furthermore, inhibition of RNMT reduces proliferation of breast cancer cells, while inducing apoptosis in HeLa cells [84, 85].

## Conclusions and future directions

In the present review, we elaborate on the physiological and pathological functions of m7G modification and the corresponding regulators in cancers. Even though research related to m7G remains in the preliminary stage, the existing studies are sufficient to suggest a crucial role of m7G in the process of tumor development. The m7G methyltransferases function to install the m7G modification at a specific location in target RNA, thus affecting the production, structure, and maturation of RNA molecules, including mRNA, miRNA, and rRNA, which ultimately regulate the translation process.

Intriguingly, regulators of the m7G modification are aberrantly expressed in various cancers and may act as novel biomarkers for diagnosis and prognostic prediction. The m7G regulator METTL1 is significantly overexpressed and promotes tumorigenesis and development in AML, BC, ESCC, glioma, HCC, HNSCC, ICC, LC, and NPC, and high expression levels of METTL1 often predict poor survival in these patients [26, 43, 52, 56, 59, 60, 66, 68, 78]. WDR4 is also highly expressed and increases malignant phenotypes in multiple malignancies, including AML, ESCC, HCC, HNSCC, IC, LC, and NPC. Increased expression of WDR4 is regarded as an unfavorable prognostic biomarker in such cancers [24, 43, 52, 59, 66, 68, 78]. In addition, METTL1 expression is associated with extremely poor prognosis in KIRC and mesothelioma, while it is related to superior survival in patients with ovarian serous cystadenocarcinoma [56]. Meanwhile, m7G regulators are also downregulated in some tumors; for example, METTL1 is downregulated in CC samples as compared with adjacent normal tissues, and WBSCR22 exhibits a lower expression level in PC as compared with peri-tumor samples [48, 80].

The m7G modification appears to serve as a double-edged sword in tumor development. The m7G regulators perform different roles in different types of tumors. For example, METTL1 and WDR4 play a strong carcinogenic role in AML, BC, ESCC, glioma, HCC, HNSCC, ICC, LC, and NPC, promoting the malignant phenotype and progression of tumors [24, 26, 43, 52, 56, 59, 60, 66, 68, 78]; however, METTL1 exerts a significant anti-cancer effect in CC [48]. WBCCR22 restrains PC development, while accelerating glioma progression [57, 80]. These m7G regulators perform biological functions by affecting m7G modification of various RNAs. The interaction between m7G modifications and various RNAs has a great impact on cancer cell growth, invasion, and metastasis. Furthermore, m7G methylation is also involved in the drug resistance of cancers. METTL1 is related to the sensitivity of chemotherapy in various cancers: overexpression of METTL1 increases the sensitivity of CC to cisplatin, while stable knockdown of METTL1 can relieve resistance to cisplatin and docetaxel in NPC and 5-fluorouracil in cervical cancer [78, 83]. In HCC, WDR4 aggravates sorafenib resistance by promoting the EMT process [61]. METTL1 and WDR4 are also associated with an immunosuppressive tumor microenvironment and participate in regulating the infiltration of various immune cells and tumor-promoting interaction between cancer cells and immune cells, which may provide potential insight into future immunotherapeutic approaches [59, 70].

**Fig. 4:**
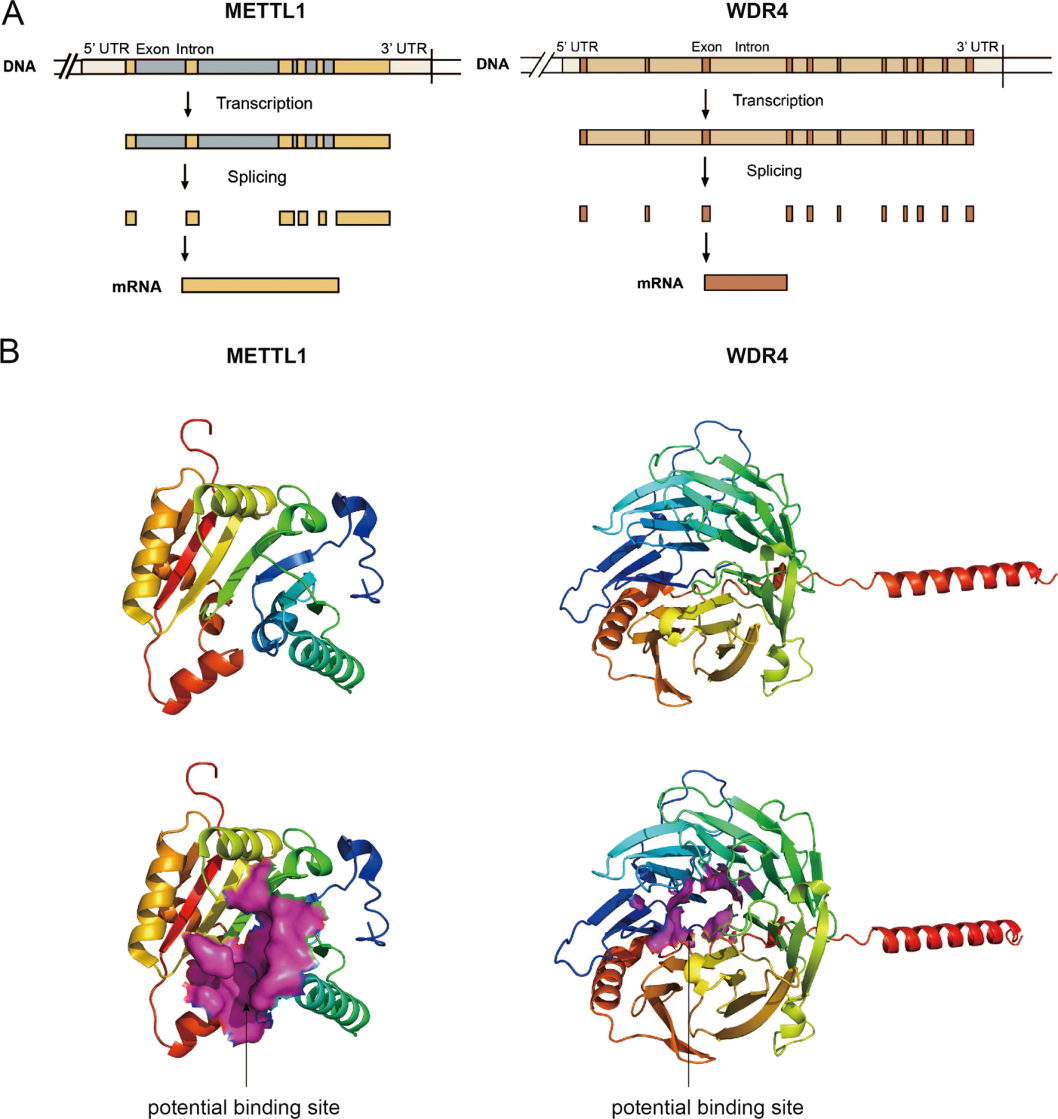
The gene and protein structures of METTL1 and WDR4. **A** The gene structures of METTL1 and WDR4. **B** The 3D structures of METTL1 and WDR4. The potential binding site was showed in rose red surface mode

The function of m7G modification in tumors has been widely explored. METTL1/WDR4, the most central regulator of m7G modification, performs an important function in a variety of tumors, exhibiting enormous potential for clinical diagnosis and treatment. Proof-of-concept studies have also revealed the role of METTL1/WDR4 in chemotherapy resistance and TIME reshaping. Targeting dysregulated METTL1/WDR4 or dysfunctional m7G sites by posttranscriptional editing may be a potential approach to eradicate tumors and may likely be combined with chemotherapy or immunotherapy to achieve better treatment efficacy in the future. Unfortunately, no METTL1/WDR4 inhibitors or potential m7G-related posttranscriptional editing systems have been reported to date. Similar to those for other RNA modifications, small-molecule inhibitors of essential m7G regulators may be the most promising and potent approach to tumor treatment; therefore, it is necessary to concentrate on the two main regulators in more detail. METTL1 is located in the 12q13 region, which includes a total of 3,635 bases and 8 transcripts. Meanwhile, the MANE Select transcript contains 6 exons (Fig. 4A). METTL1 contains an S-adenosylmethionine (SAM) binding motif that is often inactivated by phosphorylation of Ser-27 by protein kinase B (PKBα) [86]. WDR4 has been mapped to 21q22.3, which contains 36,438 bases and 7 transcripts. The MANE Select transcript includes 11 exons (Fig. 4A). WDR4 is a member of the WD repeat protein family and forms a heterodimer complex with METTL1. The protein structure of METTL1 has been unveiled; however, the structure of WDR4 remains unknown. We downloaded the METTL1 protein structure (PDB ID: 3CKK) from the RCSB Protein Data Bank (www.rcsb.org). METTL1 has 276 amino acids folded into eight α-helices and seven β-sheets (Fig. 4B). Its active pocket is composed of the residues Cys-55, Glu-77, Ile-78, Arg-79, Ser-109, Asn-110, Ala-111, Met-112, Leu-130, Phe-131, Asp-133, Thr-208, Glu-209, and Glu-210, which may be the potential binding pocket for small-molecule inhibitors. We generated a WDR4 structure using AlphaFold (www.alphafold.ebi.ac.uk), which is a novel AI system that accurately predicts the 3D structure of proteins based on their amino acid sequence. WDR4 includes 412 amino acids folded into four α-helices and twenty-eight β-sheets (Fig. 4B). It has several potential residues as inhibitor binding sites, including Leu-7, Ala-8, Leu-9, Phe-69, Thr-109, Val-151, Val-152, Val-153, and Pro-198. Meanwhile, a recent study generated a preliminary 3D model of the METTL1/WDR4 complex. This study not only analyzed the potential METTL1 SAM-binding pocket and core functional residues, but also speculated some key residues (WDR4-Arg170 and METTL1-Glu183) related to METTL1–WDR4 interaction and methyltransferase function, which may provide some useful insight for the development of METTL1/WDR4 inhibitors [55]. Further related studies are warranted to identify the actual structure of the METTL1/WDR4 complex and develop potential small-molecule inhibitors for better treatment.

In conclusion, the m7G modification is involved in a variety of physiological and pathological activities, especially oncogenesis and tumor progression; however, our understanding of m7G regulators is not yet comprehensive. Only three methyltransferase complexes, METTL1/WDR4, WBSCR22/TRMT112, and RNMT/RAM, have been identified to date, and there remain many questions regarding the intricate process of m7G modification. Firstly, it is unclear whether m7G modification is a dynamic and reversible process or whether there exist corresponding demethylases that regulate the balance of m7G modification globally. Secondly, it is unknown whether m7G methyltransferases regulate transcription by affecting the secondary structure of RNA after the addition of m7G modification or whether m7G modification provides a platform for the recruitment of corresponding m7G binding proteins to affect transcription levels. Additionally, more details are needed regarding m7G modification as an oncogenic trigger to influence translation. Several studies have demonstrated that METTL1/WDR4 negatively mediates codon-dependent ribosome pausing of m7G-modified tRNA in various malignancies. It remains unknown whether other processes associated with translation are involved in the regulation of m7G modification, for example ribosome collision-mediated translation. Finally, it is clear that m7G modification cooperates with m6Am modification to influence global RNA metabolism and translation. Since the posttranscriptional network is intricate and various regulators are often connected, it is worthwhile exploring whether m7G and other post-transcription modifications influence each other cooperatively to play a greater number of roles, especially in tumors. Further mechanistic studies are imperative to begin to unravel these mysteries.

## Data Availability

Not applicable.

## Abbreviations

AKT: AKT serine/threonine kinase 1
AML: Acute myeloid leukemia
Arg-TCT-4–1: TRNA-Arg (anticodon TCT) 4–1
BC: Bladder cancer
Bcl-2: B cell lymphoma-2
BRCA: Breast invasive carcinoma
Bud23: BUD23 rRNA methyltransferase and ribosome maturation factor
CC: Colon cancer
CCNA2: Cyclin B1
CCNB1: Cyclin B1
CCND1/2/3: Cyclin D1/2/3
CCNE1: Cyclin E1
CDK6/8: Cyclin-dependent kinase 6/8
DCP2: MRNA-decapping enzyme 2
EFEMP1: EGF-containing fibulin extracellular matrix protein 1
EGFR: Epidermal growth factor receptor
EIF2A: Eukaryotic translation initiation factor 2A
EIF4E: Eukaryotic translation initiation factor 4E
EMT: Epithelial–mesenchymal transition
ESCC: Esophageal squamous cell carcinoma
GBM: Glioblastoma
GSK3β: Glycogen synthase kinase-3 beta
HCC: Hepatocellular carcinoma
HMGA2: High mobility group AT-hook 2
HNSCC: Head and neck squamous cell carcinoma
ICC: Intrahepatic cholangiocarcinoma
IL1β: Interleukin 1 beta
IL1R2: Interleukin A receptor type 2
ISG15: Interferon-stimulated gene 15
KIRC: Kidney renal clear cell carcinoma
LC: Lung cancer
lncRNA: Long noncoding RNA
m6A_m_: *N6*, *2′-O*-Dimethyladenosine
m7G: *N*^7^-methylguanosine
m7G-MeRIP-Seq: M7G methylated RNA immunoprecipitation sequencing
m7G-Seq: M7G sequencing
m7G-miCLIP-Seq: M7G individual-nucleotide-resolution cross-linking and immunoprecipitation with sequencing
MAPK: Mitogen-activated protein kinase
METTL1: Methyltransferase-like 1
METTL14: Methyltransferase-like 14
miRNA: MicroRNA
MMP2: Matrix metallopeptidase 2
MMP9: Matrix metallopeptidase 9
mRNA: Messenger RNA
mTORC1: Mammalian target of rapamycin complex 1;
NANOG: Nanog homeobox
NPC: Nasopharyngeal carcinoma
OCT4: Octamer-binding transcription factor 4
PC: Pancreatic cancer
PCIF1: Phosphorylated CTD-interacting factor 1
PI3K: Phosphoinositide 3-kinase
PML: Promyelocytic leukemia
pri-miRNA: Primary miRNA
P-S6K: Phosphorylation of S6 kinase
qPCR: Real-time quantitative polymerase chain reaction
rRNA: Ribosomal RNA
RNMT: RNA guanine-7 methyltransferase
RAM: RNMT-activating miniprotein
RPTOR: Regulatory-associated protein of mTOR
SAA2: Serum amyloid A2
S100A4: S100 calcium-binding protein A4
snRNA: U2 small nuclear RNA
SOX2: SRY-Box transcription factor 2
Trm8p: Transfer RNA methyltransferase 8
Trm82p: Transfer RNA methyltransferase 82
TRMT112: TRNA methyltransferase activator subunit 11–2
tRNA: Transfer RNA
ULK1: Unc-51-like autophagy activating kinase 1
uPAR: Urokinase plasminogen activator surface receptor
VEGFA: Vascular endothelial growth factor A
WBSCR22: Williams–Beuren syndrome chromosome region 22
WDR4: WD repeat domain 4
